# The Role of Family or Community Nurse in Dealing with Frail and Chronic Patients in Italy: A Scoping Review

**DOI:** 10.3390/geriatrics9030081

**Published:** 2024-06-16

**Authors:** Susan Scrimaglia, Matteo Ricci, Alice Masini, Marco Montalti, Andrea Conti, Claudia Camedda, Massimiliano Panella, Laura Dallolio, Yari Longobucco

**Affiliations:** 1 Department of Biomedical and Neuromotor Sciences, Alma Mater Studiorum, University of Bologna, 40126 Bologna, Italy; scrimagliasusan@gmail.com (S.S.); matteo.ricci18@studio.unibo.it (M.R.); marco.montalti7@studio.unibo.it (M.M.); laura.dallolio@unibo.it (L.D.); 2 Department of Translational Medicine, University of Eastern Piedmont Amedeo Avogadro, 28100 Novara, Italy; andrea.conti@uniupo.it (A.C.); massimiliano.panella@uniupo.it (M.P.); 3 Doctoral Program in Food, Health, and Logevity, Department of Translational Medicine, University of Eastern Piedmont, 28100 Novara, Italy; 4 IRCCS Azienda Ospedaliero-Universitaria di Bologna, 40138 Bologna, Italy; claudia.camedda2@unibo.it; 5 Department of Medical and Surgical Sciences, Alma Mater Studiorum, University of Bologna, 40126 Bologna, Italy; 6 Department of Health Sciences, University of Florence, 50134 Firenze, Italy; yari.longobucco@unifi.it

**Keywords:** nurse, public health, scoping review

## Abstract

Even though Family and Community Nurses (FCNs) were introduced into the Italian healthcare system in 2000, to date, there is a substantial knowledge gap regarding the implementation of these professional figures within the Primary Care (PC) system. This scoping review aims to provide a comprehensive picture of the role of FCNs in managing older adults and the elderly with chronic conditions within the Italian PC system. A search on Medline, Cumulative Index to Nursing and Allied Health Literature, Cochrane Library, Embase, and Scopus was conducted including studies published until 7 April 2023. Among 141 potential articles, only 4 met our inclusion criteria. Each of these studies attributed their findings to the presence of FCNs. They reported a significant decrease in the prevalence of several behaviours among patients diagnosed with hypertension, a reduction in metabolic complications among frail patients receiving home enteral nutrition, a decline in hospital readmissions or emergency services utilization among patients >65 years of ages with at least one chronic disease, and, notably, a high level of effectiveness in detecting major cardiovascular events in patients with cardiac implantable electronic devices. Despite the effectiveness of interventions managed by FCNs, comprehensive information and research on the integration of this role within the PC setting are still lacking in the Italian healthcare system.

## 1. Introduction

Population ageing is a global phenomenon [1]. Italy, following Monaco and Japan, has the oldest population in the world [2]. In 2022, the proportion of Italians over 65 years of age stood at 24.1% [2], and it has been estimated that it will reach 35.5% by 2050 [3]. As a consequence, the prevalence of chronic conditions such as cardiovascular, respiratory, or metabolic diseases has risen as well [4]. Chronic diseases not only impose a significant burden of morbidity and mortality [5] but also account for over 80% of healthcare costs [6]. According to the Italian surveillance systems PASSI and PASSI d’Argento (Progresses in assessing population health in Italy), between 2015 and 2018, over half of the 65–75 year old population had one or two chronic conditions, with this percentage rising with age [7]. Moreover, it has been acknowledged that chronic medical conditions are closely linked to frailty [8], recognised as a multi-domain clinical condition that leads to the deterioration of physiological organ systems capacity [9].

In this scenario, addressing chronic diseases is a major challenge for healthcare systems [10], requiring the use of organisational models designed to support these categories of people defined as vulnerable [11]. Primary health care (PC) is a whole-society approach to effectively organise and strengthen national health systems to bring services for health and wellbeing closer to communities. PC was recognised by the World Health Organization (WHO) as a fundamental component of healthcare systems, whose implementation can contribute to the distribution of health and well-being by addressing health needs in individuals, families, and communities [12]. Moreover, the WHO underlined that PC could provide health promotion, prevention, treatment, and palliative care to people in need [13]. In this setting, the pivotal role of emerging figures such as Family or Community Nurses (FCNs) has been acknowledged [14]. In fact, FCNs included the independent and cooperative treatment of patients in all situations, at all ages, with families, groups, and communities, whether they are ill or not, directly in the community [14]. Nevertheless, FCNs are seen as key contributors to enhancing PC by effectively addressing the current complexity of healthcare demand while working with a multidisciplinary team [15]. Additionally, the core competencies and specific skills of FCNs have been clearly identified and defined by the European Curriculum for Family and Community Nurse [16,17]. The COVID-19 pandemic has further emphasised the urgency of implementing PC models to meet the growing demand for care [18]. Therefore, FCNs were massively introduced in Italy in 2020 [19]. Two years later, their role was framed within the models and standards for the development of Italian territorial healthcare assistance (DM 77/2022). The Italian National Prevention Plan 2020–2025 [20] officially recognized the role of FCNs for the first time. In order to clearly suggest the potential implications of this figure, the National Agency for Regional Health Services (AGENAS) published the “Guidelines for Family or Community Nurses” [21]. In this document, FCNs are designated with responsibilities encompassing promotion, prevention, and health management for individuals, caregivers, and the community [22]. However, it is worth mentioning that while FCNs have only recently been clearly characterized, experimental projects involving this type of figure had already been introduced in Italy [23], aligning with practices in other European countries [17]. This scoping review arises from the need to assess whether the role of FCNs in Italian PC settings has been investigated and aligns with the established guidelines. Despite numerous experiences documented in the literature regarding FCN projects, a comprehensive summary of FCNs’ involvement and activities in PC settings has yet to be undertaken. The aim of our scoping review is to provide a current and comprehensive picture of the role of FCNs in managing frail and chronic patients within the Italian PC system. Moreover, this work seeks to summarise available information on FCN-led interventions, tasks, and activities and the types of chronic conditions they address and manage.

## 2. Materials and Methods

### 2.1. Study Design

The present scoping review [24] was conducted following the methodological framework outlined by Arksey and O’Malley [25]. Moreover, the review was reported according to the PRISMA extension for Scoping Reviews (PRISMA-ScR) [26].

### 2.2. Research Question

To guide the search strategy, the following review question was identified: “What is the current knowledge on Family and Community Nurse in Italy in dealing with frail or chronically ill patients?”. Specifically, we asked the following: (i) “What roles do FCNs currently have?” and (ii) “Which interventions, tasks, or activities do FCNs undertake?”

### 2.3. Relevant Studies and Selection Criteria

Eligibility criteria were established through the Population, Concept, and Content framework (PCC) using the following research items: population: nurse; concept: frail or chronic disease; content: Italy.

Criteria of inclusion and exclusion are described in Table 1.

The above inclusion and exclusion criteria were established based on the need to investigate the role of the FCNs in PC settings in dealing with chronic and frail patients.

### 2.4. Search Study

Based on the PCC framework, SS and YL developed a comprehensive search strategy which combined MeSH terms, Boolean operators, and appropriate wildcards to account for plurals or variations in spelling. The search string was (“Community Nurs*” OR “Community Health Nurs*” OR Nurs*, Community Health OR “Home Health Nurs*” OR Nurs*, Home Health OR “Home Nurs*” OR “Family Nurse Practition*” OR Nurse Practition*, Family OR “Family Nurs*” OR Nurs*, Family OR “Family-Centered Nurs*” OR “Family Centered Nurs*” OR Nurs*, Family-Centered OR “Nursing, Community Health” OR “family health nurs*” OR Infermie* di famiglia OR Infermie* di comunità OR Infermie* di famiglia e di comunità OR infermie* di famiglia o di comunità OR Infermie* di famiglia e comunità OR Infermie* di famiglia o comunità) AND (Frailty OR Frailties OR Frailness OR “Frailty Syndrome” OR Aging OR Aged OR Elderly OR “Frail Older Adul*” OR “Older Adul*” OR “Older Person” OR “Older People” OR Anzian* OR Fragile OR Fragilità OR Anziano Fragile OR Anziani Fragili OR “Functional Geriatric Evaluation” OR “Functional Geriatric Assessment” OR “Comprehensive Geriatric Assessment” OR Valutazione Geriatrica Multidimensionale OR Valutazione della Funzionalità Geriatrica OR “Pro-Active Care” OR “Proactive Care” OR Assistenza Proattiva) AND (Italy [text word] OR Italian [text word] OR Italia [text word] OR Italiano [text word] OR Italian Region [text word] OR Italian Context [text word]). When necessary, the search string was adapted to perfectly fit in each database. The search was conducted on 7 April 2023, consulting Medline (PubMed), Cumulative Index to Nursing and Allied Health Literature (CINAHL), Cochrane Library, Embase, and Scopus databases.

### 2.5. Screening and Study Selection

Duplicate records were removed, and titles and abstracts were independently screened by seven researchers (YL, LD, AM, MM, MR, CC, and SS). Then, full texts of relevant articles were retrieved and independently assessed by eight researchers (YL, LD, AM, MM, MR, SS, CC, and AC). Disagreements between authors were solved through discussion and consensus between reviewers.

### 2.6. Data Extraction

Four researchers (CC, MM, MR, and SS) independently extracted and summarised information using a standardised form [27], which included the following information: digital object identifier, title, first author, publication year, study design, period of study, study aim, type of nurse described, description of the nurse’s role, setting of work, patients characteristics, interventions, outcomes, and results. Then, information was double-checked by a second researcher.

### 2.7. Quality Assessment and Risk of Bias

While scoping reviews do not typically appraise the methodological quality, the Authors decided to assess the risk of bias of the included studies. In detail, methodological quality was assessed using the Joanna Briggs Institute (JBI) Critical Appraisal Checklists for Cohort Studies [28], Quasi Experimental Studies [29], and Randomized Controlled Trials [30]. Each checklist was assessed by multiple questions (i.e., “Was true randomization used for assignment of participants to treatment groups?” “Were treatment groups similar at the baseline?” “Were outcomes measured in a reliable way?”), for which the possible answers were “yes”, “no”, “unclear”, or “not applicable”. Articles were evaluated based on the following criteria that, as recommended by the JBI manual, were decided on and approved by all the authors: (i) “High quality” if all the criteria were met; (ii) “Medium quality” if one or more criteria were unclear; (iii) “low quality” if one or more criteria were not met. Four researchers (LD, YL, AM, and SS) independently assessed the quality of included studies. Conflicts in the quality scores were resolved through discussion and consensus between the researchers.

## 3. Results

### 3.1. Search Results

**Figure 1:**
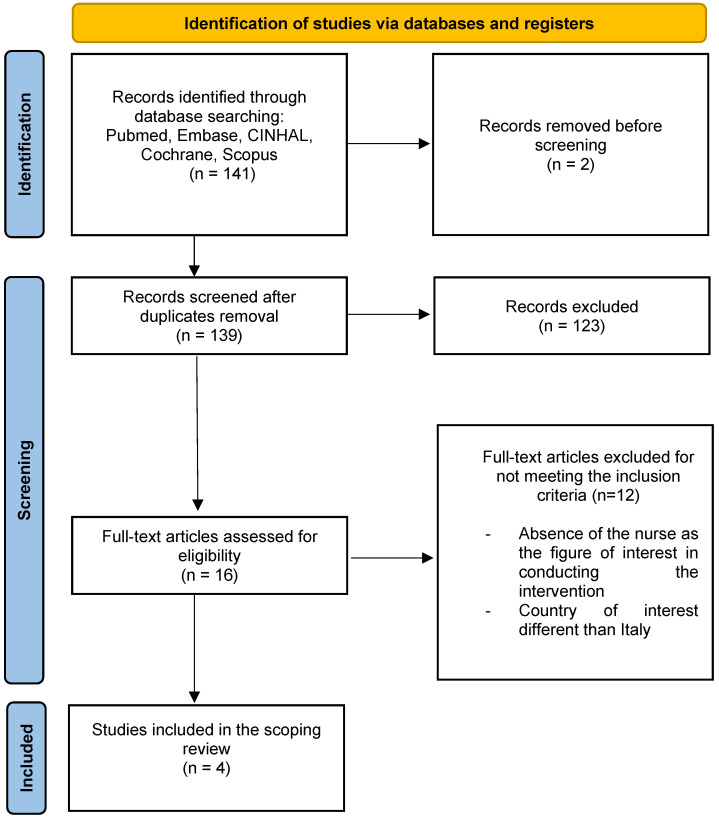
PRISMA-ScR diagram.

The search identified 141 potentially relevant records. After the removal of 2 duplicates, 123 articles were excluded based on title, abstract, and/or a portion of the text. A total of 16 were eligible for full-text reading. Finally, 4 papers were included, as they fulfilled the established criteria. The PRISMA-ScR diagram is shown in Figure 1. The main reasons for exclusion were countries of interest different than Italy and the absence of the figure of the nurse.

### 3.2. Study Characteristics and Data Extraction

Data on the included studies are shown in Table 2. Of the included papers, two were randomised controlled trials (RCTs) [31,32], one was a quasi-experimental pilot study [33], and one was a prospective study [34]. The included studies had a duration of 7 to 8 months, and all of them investigated the role of FCNs in dealing with chronic/frail patients. The quasi-experimental pilot study by Savini et al. [33] aimed to assess whether a structured FCN-led educational intervention was effective in reducing hospital readmissions or emergency services’ use in patients over 65 years with at least one chronic illness (excluding cancer and/or terminal disease). The intervention was conducted in PC services by a certified FCN. In detail, it consisted of weekly, face-to-face, 60 min sessions aimed to target aspects of the patient’s disease, medication adherence, treatment, and health behaviours. The sessions were designed following the teach-back approach to assess the education. Specifically, this educational intervention was based on asking the patient to repeat the instruction they received to increase their understanding of the information delivered by the nurses. The main activities led by the FCNs consisted of assessing patients’ educational needs, delivering nursing diagnosis, and educating and empowering patients. Results showed a decrease in emergency services’ use after the tailored intervention and an increase in the patient’s satisfaction.

Ricci et al. [34] conducted a prospective cohort study introducing a novel organizational model centred on “Primary Nursing”, specifically tailored to remote monitoring. In this study, the roles and responsibilities of every member of the team involved in monitoring patients with cardiac implantable electronic devices were defined. Specifically, FCNs had to enter patients’ data in electronic systems and schedule clinical alerts and periodical transmissions of information according to individual clinic profiles. If an alert occurred, the FCN received an email and then reviewed and screened the patients’ clinical conditions in agreement with written protocols and contacted the patient for clinical information. Phone contact was used by the FCN to monitor drug therapy compliance, long term clinical status, and patient recall in case of missed transmission. Moreover, bilateral communication was possible, as patients were encouraged to contact the nurse for clinical assistance. This FCN-based model showed a high effectiveness in detecting major cardiovascular events.

Cicolini et al. [32] tested in their RCT the efficacy of individualised FCN-led lifestyle educational programs in improving blood pressure and adherence to lifestyle recommendations in patients with a diagnosis of hypertension. The nurse care manager coordinated follow-up visits, recorded data, and carried out educational programs. In the intervention group, the nurse also sent weekly email phone reminders on healthy lifestyle behaviours, also containing recommendations on diet, exercise, smoking cessation, alcohol consumption, self-pressure monitoring, and medication adherence. In conclusion, the prevalence of several behaviours or conditions of risk decreased significantly more than in the control group.

The RCT by Orlandoni et al. [31] evaluated outcomes of home enteral nutrition in frail elderly patients by using video consultation between monthly dedicated home visits. The home visiting staff, which included FCNs, visited each patient monthly to carry out examination, assessment, and the prevention/management of complications. In the intervention group, FCNs carried out a video consultation with the hospital physician during home visits, who visually examined the patient. Results showed a reduction in metabolic complications.

### 3.3. Quality Assessment

The observational prospective cohort study performed by Ricci et al. was scored as good quality [34]. The RCT by Cicolini et al. was classified as medium quality mainly due to the unclear information related to the blindness of the evaluators [32]. The RCT by Orlandoni et al. was classified as a low level of quality considering the lack of allocation concealment [31]. The quasi-experimental study conducted by Savini et al. was scored as good quality [33]. The summary of the risk of bias assessment is displayed in Table 3.

## 4. Discussion

The aim of the present scoping review is to summarise the role of FCNs in dealing with chronic and frail patients within the Italian PC setting. Our review compiles the available information concerning the interventions managed by the FCN on frail, chronic, or elderly individuals in the Italian PC setting.

The interventions were primarily conducted at home and required home visits [31,32,33,34]. These findings are aligned with the growing need to provide home health care services as an essential component of PC services [35]. There is evidence that home visits, which deliver clinical services directly to patients in their community, have positive effects on health outcomes [36]. For example, the study conducted by Pooresmaeil et al. claims that home visiting programs significantly improve the level of knowledge of haemodialysis patients and their families through the involvement and implementation of caregivers and patients in the care process and in the standard care plans [37]. Conversely, the study by Friedman et al. highlights that home nursing visits can help older people in managing multiple daily activities (ADLs) [38]. As confirmed by our results, a recent umbrella review found that hospital admissions for older people have decreased following nursing home visits [39]. Furthermore, it has been suggested that home care is the most cost-effective way to increase access to healthcare services for vulnerable people [35].

Among all the included studies, the nurses maintained professional relationships with colleagues and worked in multidisciplinary teams [31,32,34]. This is consistent with the increasing emphasis on the advantages of working in teams in terms of work satisfaction and improving patients’ health outcomes [40].

The use of telemedicine systems was implemented in two included studies [31,32,34]. Over the last decade, the literature in the field has grown [41], and the use of telemedicine has become more frequent.

In summary, the benefits of introducing this figure into the PC system are clearly identified by current knowledge.

The ENhANCE project (EuropeaN curriculum for fAmily aNd Community nurse defined the standardised professional profile of the European FCN, based on specific core competencies adopted two years later by the Italian Nursing Order in a positional statement. In line with this important document, our scoping review outlined some of these core competencies in the included studies, such as identifying and assessing an individual’s health status and health needs [31,33], monitoring the patient’s health [31,34], applying education strategies, and promoting health [32,33,34].

Caregiving is a dynamic process, and a periodic assessment of health needs is important to identify issues and suggest strategies for improving future assistance [42]. There has been an increased emphasis on the importance of assessing patients’ needs, and nurses are implementing their assessment skills [43]. Moreover, in accordance with the main core competencies, FCNs are essential figures in embedding health promotion initiatives [44]. In this regard, the literature widely documents the effectiveness of nurse-led educational strategies on the health outcomes of frail and chronic patients [45,46,47,48,49,50].

### 4.1. Study Limitation

Our results show some important limitations as well as opportunities for improvement. In all four articles, certain skills related to addressing family and community needs were not emphasized. The possible reason for this finding could be that three out of four articles were published before the development of the FNC curriculum by the ENhANCE project [31,32,34]. An observational study conducted in 2022, involving over 50,000 patients, showed that the information recorded by nurses using an assessment framework based on health patterns and standardised nursing languages was useful in describing the profile of highly complex chronic patients in PC, allowing for a stratification of care profiles in a specific healthcare area [51]. However, this approach was not applied to any of the included studies. Patients had specific chronic diseases, such as hypertension, heart disease, and a need for enteral nutrition, which were not exhaustively addressed. Moreover, the duration of each study was relatively short; studies with long-term follow-up would be useful to evaluate the efficacy and cost–benefit ratio of nursing interventions.

Finally, the generalisability of our scoping review presented some limitations due to the small numbers of included studies which were performed only in the Italian context, even though we were interested in providing a comprehensive summary of the role of FCNs in national PC settings.

### 4.2. Application to Practise

Health policies are encouraging the implementation of this figure in Italian PC settings by considering their capacity in managing the main health problems and the factors influencing them and their capacity in identifying the community’s resources, e.g., the involvement and empowerment of the people belonging to the community [52].

Experimental projects have been undertaken in some Italian areas, such as the Habitat project in the Friuli-Venezia Giulia Region, the TESEO project in the Emilia-Romagna Region, or the FCN project in Tuscany [53]. However, the evidence on this topic is currently heterogeneous and fragmented, with scientific data still being insufficient. An exception is the Community Nurse Supporting Elderly in a Changing Society (CONSENSO) project, a European initiative conducted across various European regions, including two regions in Italy, and aimed at developing a care model where the elderly could maintain their functional capacity [54]. Despite the lack of some information (e.g., the absence of an FCN implementation framework), these results are encouraging.

Therefore, in our review, it was not possible to identify a unique model that can be applied nationally. Several governments, including the Italian one, have adopted policies explicitly acknowledging FCNs. International literature shows emerging evidence on FCN implementation, in particular in the UK. Dellafiore et al. attempted to provide an overview of FCNs; however, due to the methodological heterogeneity of the included articles, most of which were conducted in the UK, it was not feasible to provide a unified definition of FCNs [55]. Many European countries are still in the initial phases of implementing FCNs [56]. Moreover, nurse practices, competencies, and health organisation vary from country to country; therefore, it is difficult to apply one single model. National and regional documents describe the need for implementing this figure; however, to date, clear examples to follow are lacking. For this reason, the consolidation of the FCN is still a work in progress. In our opinion, in order to achieve an effective model, it is necessary to adequately assess the local context, creating a framework that describes local needs and resources. This could be beneficial to determine priority areas of intervention and to define the activities that the FCN could manage in specific settings. It is mandatory that FCN roles and responsibilities are clearly defined, and that local FCN models are structured to prevent overlaps with other professional roles, ensure efficient task allocation, and avoid redundant service provision. To the best of our knowledge, this is the first scoping review that synthesises the available evidence on the role of FCNs in the Italian context. To date, as suggested by the limited number of articles found, Italian FCN models have not received enough attention; therefore, research should move toward defining practice models that are effective in local contexts.

## 5. Conclusions

Epidemiologic and demographic shifts leading to increased care demands coupled with diminished economic resources pose formidable challenges to public health. As a result, the development of new organisational models aimed at enhancing the care of chronic and frail patients is imperative. Primary healthcare emerges as an effective approach to ensure high levels of health, with the FCN recognised as a pivotal figure for implementing these models. This scoping review includes initial experiences with healthcare models that involve nurses in the management of chronic and frail patients within Italian PC settings, where the integration of this figure is progressing with variability. Studies focused on FCNs can offer valuable insights to guide policies and stakeholder decisions regarding the implementation and support of the FCN’s role. Nevertheless, given the limited number of existing studies, further research in this field is warranted.

## Figures and Tables

**Table 1:** Inclusion/exclusion criteria based on PCC.

Parameter	Inclusion Criteria	Exclusion Criteria
Population	Articles describing nurses’ roles, tasks, and activities	Paediatric and school nurses
Concept	Frail and/or chronic patients	
Context	Primary health care	Settings different to primary care one (e.g., hospitals, inpatient clinics)
	Country of interest: Italy	
Study Design	Primary research (both experimental and observational studies) based on original data	Language different from English or Italian; studies published more than 10 years ago

**Table 2:** Data extraction.

Author; Year	Study Design; Duration	Setting	Population	FCN Background and Role	FCN Tasks and Responsibilities	Study Tasks
Ricci R. et al. [34]; 2013	Prospective cohort study; 8 months (mean follow-up)	Patient’s home	Patients that had received pacemakers and ICD equipped with the wireless Biotronik Home MonitoringTM function	Nurse responsible of the continuity of care, based on the “Primary Nursing” model	(i) Training and education; (ii) Website data entry; (iii) Remote data review; (iv) Data screening; (v) Critical case submission to physician; (vi) Contacting patients; (vii) Checking patient compliance and therapy benefits.	(i) Patient education of benefits and limitations of telemedicine; (ii) Patients’ data input and clinical alert scheduling; (iii) Alerts monitoring and reviewing; (iv) Therapy adherence telephone monitoring;
Cicolini G. et al. [32]; 2014	Randomized controlled trial; 7 months.	(i) Patient’s home; (ii) Hypertension Primary Care Centre.	Patients on active treatment for hypertension, or with systolic blood pressure ≥ 140 mmHg, or with diastolic blood pressure ≥ 90 mmHg	Care Manager Nurse	(i) Coordination of follow-up visits; (ii) Record of baseline and follow-up data using structured forms; (iii) Educational program carrying out; (iv) Send reminders.	Usual Care: (i) Routine follow-up visits at 1, 3 and 6 months after enrollment; (ii) Patients were invited to inform the nurses of drug adherence and follow an educational program. A 1 h session, in which the nurses discussed the importance of blood pressure control and correct measurement (giving advice for a correct blood pressure self-measurement). In addition, nurses reported non-pharmacological strategies for a healthy lifestyle, and instruction on completing the daily self-assessment form. Intervention: (iii) Patients also received phone calls and email alerts from the nurses; (iv) Phone calls and email follow-up; (v) Weekly email containing evidence-based lifestyle interventions
Orlandoni P. et al. [31]; 2016	Randomized controlled trial; 12 months	Patient’s home	Patients ≥65 years receiving home enteral nutrition from the Department of Clinical Nutrition of an Italian geriatric hospital	Nurse that worked as home visiting staff along with the physician	(i) Assessment of the patient; (ii) Nursing diagnosis and management of complications; (iii) Encourage video consultation.	Regular home visits: (i) Monthly scheduled assessment (i.e., ECG, pulse oximetry, dysphagia assessment, nutritional status, etc.); (ii) Diagnosis and management of tube related complications (e.g., tube displacement or occlusion); (iii) Gathering of additional relevant information about the patients (e.g., if the patient had additional and independent medical examinations). Intervention: Video consultation with a clinical nutrition physician during home visits.
Savini S. et al. [33]; 2021	Quasi-experimental pilot study; 8 months.	(i) General practices that cover primary care; (ii) Patients’ home.	Adults ≥65 years. Patients with at least one chronic condition (illness lasting more than 6 months), with the exclusion of patients with important neurological and/or cognitive deficits, terminal disease and/or cancer.	Certified Family Nurse Practitioner (FNP), trained with a 12 h “teach-back education” course and with one year of experience.	(i) Home care visits (vital signs check; medication, drug administration, support in daily living); (ii) Patient educational needs assessment; (iii) Nursing diagnosis (based on Clinical Care Classification System); (iv) Tailored patient education; (v) Patients’ empowerment.	Before the intervention: (i) Bi-weekly home care visits including the provision of functional support during activities of daily living, medications, vital signs check, IV drug administration, blood samples taking. During the intervention: (ii) Initial patients’ educational need assessment; clinical care classification-based nursing diagnosis; (iii) Addressing of self-management abilities; (iv) Delivery of a weekly, face-to-face, “teach back approach”-based educational intervention. The intervention aimed at targeting aspects of the disease and its treatment, potential complications, medical adherence and health behaviours; (v) Patients’ encouragement to improve their self-management behaviours.

**Table 3:** Quality assessment of included studies.

Authors	Study Design	Tool for Assessment	Quality
Ricci et al., 2013 [34]	Cohort study	The JBI Critical Appraisal Checklist for Cohort Studies	Good
Cicolini et al., 2014 [32]	RCT	The JBI Critical Appraisal Tool for RCTs	Medium quality
Orlandoni et al., 2016 [31]	RCT	The JBI Critical Appraisal Tool for RCTs	Low level of quality
Savini et al., 2021 [33]	Quasi-experimental	The JBI Critical Appraisal Tool for Quasi-experimental	Good

RCT: Randomized control trial, JBI: Joanna Brings Institute.

## Data Availability

The data used to support the findings of this study are available from the corresponding author upon request.

## References

[1] Bloom D.E., Luca D.L. (2016). The global demography of aging: Facts, explanations, future. Handbook of the Economics of Population Aging.

[2] ISTAT Indicatori Demografici—La Popolazione Cala Ancora ma non al Livello del Biennio 2020-21. Aumentano Gli Stranieri 2022. https://www.istat.it/it/files//2023/04/indicatori-anno-2022.pdf.

[3] Galluzzo L., Gandin C., Ghirini S., Scafato E. Population Ageing: Opportunity or Challenge?. https://www.epicentro.iss.it/ben/2012/aprile/2.

[4] Atella V., Piano Mortari A., Kopinska J., Belotti F., Lapi F., Cricelli C., Fontana L. (2019). Trends in age-related disease burden and healthcare utilization. Aging Cell.

[5] Probst-Hensch N., Kunzli N. (2012). Preventing noncommunicable diseases-beyond lifestyle. Epidemiology.

[6] Holman H.R. (2020). The Relation of the Chronic Disease Epidemic to the Health Care Crisis. ACR Open Rheumatol..

[7] Masocco M., Minardi V., Contoli B., Bertozzi N., Campostrini S., Carrozzi G., Cristofori M., D’Argenzio A., De Luca A.M.C., Fateh-Moghadam P. Patologie Croniche Nella Popolazione Residente in Italia Secondo i Dati PASSI e PASSI d’Argento. https://www.epicentro.iss.it/coronavirus/sars-cov-2-flussi-dati-confronto-passi-pda-cronicita#writers.

[8] Mangin D., Lawson J., Risdon C., Siu H.Y., Packer T., Wong S.T., Howard M. (2023). Association between frailty, chronic conditions and socioeconomic status in community-dwelling older adults attending primary care: A cross-sectional study using practice-based research network data. BMJ Open.

[9] Dent E., Martin F.C., Bergman H., Woo J., Romero-Ortuno R., Walston J.D. (2019). Management of frailty: Opportunities, challenges, and future directions. Lancet.

[10] Reynolds R., Dennis S., Hasan I., Slewa J., Chen W., Tian D., Zwar N. (2018). A systematic review of chronic disease management interventions in primary care. BMC Fam. Pract..

[11] World Health Organization, Noncommunicable Disease, and Mental Health Cluster (2002). Innovative Care for Chronic Conditions: Building Blocks for Actions: Global Report.

[12] WHO (United Nations Children’s Fund) (2022). Primary Health Care Measurement Framework and Indicators: Monitoring Health Systems through a Primary Health Care Lens.

[13] WHO Primary Health Care 2021. https://www.who.int/news-room/fact-sheets/detail/primary-health-care.

[14] WHO (Regional Office for Europe) (1999). HEALTH21: The Health for All Policy Framework for the WHO European Region.

[15] Marcadelli S., Stievano A., Rocco G. (2019). Policy proposals for a new welfare: The development of the family and community nurse in Italy as the key to promote social capital and social innovation. Prim. Health Care Res. Dev..

[16] Pozzi F., Passarelli M., Manganello F., Alvino S., Dagnino F., Mazzarino B., Rodrigues C. (2021). Development of a European Curriculum for Family and Community Nurses.

[17] Camedda C., Scalorbi S., Longobucco Y. (2021). The Family and Community Nurse Core Competencies: Integrating European standards into the Italian context. Acta Biomed..

[18] Rawaf S., Allen L.N., Stigler F.L., Kringos D., QuezadaYamamoto H., van Weel C., Global Forum on Universal Health Coverage and Primary Health Care (2020). Lessons on the COVID-19 pandemic, for and by primary care professionals worldwide. Eur. J. Gen. Pract..

[19] Ministero Dell’ Istruzione DECRETO 71 del 25 Luglio 2020. https://www.miur.gov.it/-/decreto-ministeriale-n-71-del-25-luglio-2020.

[20] Ministry of Health I. (2020). National Prevention Plan 2020-25. https://www.emcdda.europa.eu/drugs-library/ministry-health-italy-2020-national-prevention-plan-2020-25_en.

[21] AGENAS Agenas Pubblica le Linee di Indirizzo Infermiere di Famiglia o Comunità 2023. https://www.agenas.gov.it/comunicazione/primo-piano/2298-agenas-pubblica-le-linee-di-indirizzo-infermiere-di-famiglia-o-comunità.

[22] Ministero Dell’ Istruzione DECRETO 23 Maggio 2022, n. 77 2022. https://www.gazzettaufficiale.it/eli/id/2022/06/22/22G00085/sg.

[23] Camedda C., Valcavi L., Saccaggi L.L., Guberti M. (2022). L’Infermierə di Famiglia e di Comunità. Infermieristica Preventiva, di Famiglia e di Comunità.

[24] Munn Z., Peters M.D.J., Stern C., Tufanaru C., McArthur A., Aromataris E. (2018). Systematic review or scoping review? Guidance for authors when choosing between a systematic or scoping review approach. BMC Med. Res. Methodol..

[25] Arksey H., O’Malley L. (2005). Scoping studies: Towards a methodological framework. Int. J. Soc. Res. Methodol..

[26] Tricco A.C., Lillie E., Zarin W., O’Brien K.K., Colquhoun H., Levac D. (2018). PRISMA Extension for Scoping Reviews (PRISMA-ScR): Checklist and Explanation. Ann. Intern. Med..

[27] Torrens C., Campbell P., Hoskins G., Strachan H., Wells M., Cunningham M., Maxwell M. (2020). Barriers and facilitators to the implementation of the advanced nurse practitioner role in primary care settings: A scoping review. Int. J. Nurs. Stud..

[28] Moola S., Munn Z., Tufanaru C., Aromataris E., Sears K., Sfetcu R., Mu P.F. (2020). Systematic reviews of etiology and risk. Joanna Briggs Institute Reviewer’s Manual.

[29] Tufanaru C., Munn Z., Aromataris E., Campbell J., Hopp L., Aromataris E., Lockwood C., Porritt K., Pilla B., Jordan Z. (2024). Systematic reviews of effectiveness (2020). JBI Manual for Evidence Synthesis.

[30] Barker T.H., Stone J.C., Sears K., Klugar M., Tufanaru C., Leonardi-Bee J., Munn Z. (2023). The revised JBI critical appraisal tool for the assessment of risk of bias for randomized controlled trials. JBI Evid. Synth..

[31] Orlandoni P., Jukic Peladic N., Spazzafumo L., Venturini C., Cola C., Sparvoli D., Fagnani D. (2016). Utility of video consultation to improve the outcomes of home enteral nutrition in a population of frail older patients. Geriatr. Gerontol. Int..

[32] Cicolini G., Simonetti V., Comparcini D., Celiberti I., Di Nicola M., Capasso L.M., Manzoli L. (2014). Efficacy of a nurse-led email reminder program for cardiovascular prevention risk reduction in hypertensive patients: A randomized controlled trial. Int. J. Nurs. Stud..

[33] Savini S., Iovino P., Monaco D., Marchini R., Di Giovanni T., Donato G., Turci C. (2021). A family nurse-led intervention for reducing health services’ utilization in individuals with chronic diseases: The ADVICE pilot study. Int. J. Nurs. Sci..

[34] Ricci R.P., Morichelli L. (2013). Workflow, time and patient satisfaction from the perspectives of home monitoring. Europace.

[35] WHO (Regional Office for the Eastern Mediterranean) (2015). The Growing Need for Home Health Care for the Elderly (Home Health Care for the Elderly as an Integral Part of Primary Health Care Services). https://applications.emro.who.int/dsaf/EMROPUB_2015_EN_1901.pdf?ua=1.

[36] Fagerström L., Wikblad A., Nilsson J. (2009). An integrative research review of preventive home visits among older people--is an individual health resource perspective a vision or a reality?. Scand. J. Caring Sci..

[37] Pooresmaeil M., Iranpour S., Aghamohammadi M. (2023). Effects of a nurse-led structured home visiting program on quality of life and adherence to treatment in hemodialysis patients. Front. Public Health..

[38] Friedman B., Li Y., Liebel D.V., Powers B.A. (2014). Effects of a home visiting nurse intervention versus care as usual on individual activities of daily living: A secondary analysis of a randomized controlled trial. BMC Geriatr..

[39] Eltaybani S., Kawase K., Kato R., Inagaki A., Li C.C., Shinohara M., Sumikawa Y. (2023). Effectiveness of home visit nursing on improving mortality, hospitalization, institutionalization, satisfaction, and quality of life among older people: Umbrella review. Geriatr. Nurs..

[40] Smolowitz J., Speakman E., Wojnar D., Whelan E.M., Ulrich S., Hayes C., Wood L. (2015). Role of the registered nurse in primary health care: Meeting health care needs in the 21st century. Nurs. Outlook.

[41] Rasmussen B.S., Jensen L.K., Froekjaer J., Kidholm K., Kensing F., Yderstraede K.B. (2015). A qualitative study of the key factors in implementing telemedical monitoring of diabetic foot ulcer patients. Int. J. Med. Inform..

[42] Levine C. (2011). Supporting family caregivers: The hospital nurse’s assessment of family caregiver needs. Am. J. Nurs..

[43] Lesa R., Dixon A. (2007). Physical assessment: Implications for nurse educators and nursing practice. Int. Nurs. Rev..

[44] Phillips A. (2019). Effective approaches to health promotion in nursing practice. Nurs. Stand..

[45] Qu X., Shen P. (2023). Investigating polymorphisms related to chronic kidney disease and the effect of health and nursing education on self-management ability and quality of life in hemodialysis patients. Cell. Mol. Biol..

[46] Hu W., Li T., Cao S., Gu Y., Chen L. (2022). Influence of Nurse-Led Health Education on Self-Management Ability, Satisfaction, and Compliance of Elderly Patients with Chronic Obstructive Pulmonary Disease Based on Knowledge, Belief, and Practice Model. Comput. Math. Methods Med..

[47] Alves A.M., Rodrigues A., Sa-Couto P., Simoes J.L. (2021). Effect of an Educational Nursing Intervention on the Mental Adjustment of Patients with Chronic Arterial Hypertension: An Interventional Study. Int. J. Environ. Res. Public Health.

[48] Cui X., Zhou X., Ma L.L., Sun T.W., Bishop L., Gardiner F.W., Wang L. (2019). A nurse-led structured education program improves self-management skills and reduces hospital readmissions in patients with chronic heart failure: A randomized and controlled trial in China. Rural. Remote Health.

[49] Gorina M., Limonero J.T., Alvarez M. (2018). Effectiveness of primary healthcare educational interventions undertaken by nurses to improve chronic disease management in patients with diabetes mellitus, hypertension and hypercholesterolemia: A systematic review. Int. J. Nurs. Stud..

[50] Chan S.S., Leung D.Y., Leung A.Y., Lam C., Hung I., Chu D., Yuen K.Y. (2015). A nurse-delivered brief health education intervention to improve pneumococcal vaccination rate among older patients with chronic diseases: A cluster randomized controlled trial. Int. J. Nurs. Stud..

[51] Brito-Brito P.R., Rodriguez-Alvaro M., Fernandez-Gutierrez D.A., Martinez-Alberto C.E., Cabeza-Mora A., Garcia-Hernandez A.M. (2022). Nursing Diagnoses, Planned Outcomes and Associated Interventions with Highly Complex Chronic Patients in Primary Care Settings: An Observational Study. Healthcare.

[52] Pazzaglia C., Camedda C., Ugenti N.V., Trentin A., Scalorbi S., Longobucco Y. (2023). Community Health Assessment Tools Adoptable in Nursing Practice: A Scoping Review. Int. J. Environ. Res. Public Health.

[53] Scalorbi S., Longobucco Y., Trentin A. (2022). Infermieristica Preventiva, di Famiglia e di Comunità.

[54] CO.N.S.E.N.SO (2021). Community Nurse Supporting Elderly in a Changing Society. https://www.alpine-space.eu/project/co-n-s-e-n-so/.

[55] Dellafiore F., Caruso R., Cossu M., Russo S., Baroni I., Barello S., Arrigoni C. (2022). The State of the Evidence about the Family and Community Nurse: A Systematic Review. Int. J. Environ. Res. Public Health.

[56] Bagnasco A., Catania G., Zanini M., Pozzi F., Aleo G., Watson R., Hayter M., Sasso L. (2022). ENHANCE WP2 Collaborative Group. Core competencies for family and community nurses: A European e-Delphi study. Nurse Educ. Pract..

